# Usefulness of Topical Imiquimod 3.75% in Cytokeratin 7 Positive Extramammary Paget Disease of the Vulva: Towards Personalized Therapy

**DOI:** 10.5826/dpc.1102a11

**Published:** 2021-03-08

**Authors:** Sara Mazzilli, Annunziata Dattola, Anna Angela Criscuolo, Terenzio Cosio, Luca Bianchi, Elena Campione, Monia Di Prete, Elisabetta Botti

**Affiliations:** 1 Dermatology Unit, Department of Systems Medicine, University of Rome Tor Vergata, Italy; 2 Department of Gynecology, University of Rome Tor Vergata, Italy; 3 Anatomical Pathology, University of Rome Tor Vergata, Italy

**Keywords:** imiquimod, extramammary Paget disease, vulva, cytokeratin 7

## Introduction

Extramammary Paget disease (EMPD) of the vulva, a rare adenocarcinoma in situ, is often resected with involved margins. The clinical presentation presents with erythematous plaques of sluggish growth, well-defined edges, fine scaling, excoriations, ulcerations, and lichenification. EMPD can manifest as a single whitish or confluent, sometimes ulcerated lesion. Candidiasis, psoriasis and chronic lichen simplex should be considered in the differential diagnoses [1]. Immunohistochemistry studies became the gold standard for pathologic prognosis, and the data for EMPD includes cytokeratin 7 (CK7) that appears to be related to slow pathologic progression [2]. The histogenesis of EMPD is complex; Paget cells could originate from the underlying intraductal cancer and migrate through the basement membrane to the nipple or genital areas. Histopathology represents the gold standard for diagnosis of EMPD and immunohistochemical staining differentiates between this skin disorder and intraductal cancer. It is postulated that mammary Paget disease as an in-situ carcinoma with Paget cells undergoing malignant transformation. Surgical excision and micrographic surgery are generally the best treatment options, although recurrences are frequent because it is difficult to obtain optimal surgical margins. Local recurrence rates after surgery vary from 34% to 56%, and patients often experience a real “surgical calvary.” In cases of very extensive lesions or difficult locations, such as the genital localization, second-line therapy such as topical therapy could be proposed.

## Case Presentation

**Figure 1:**
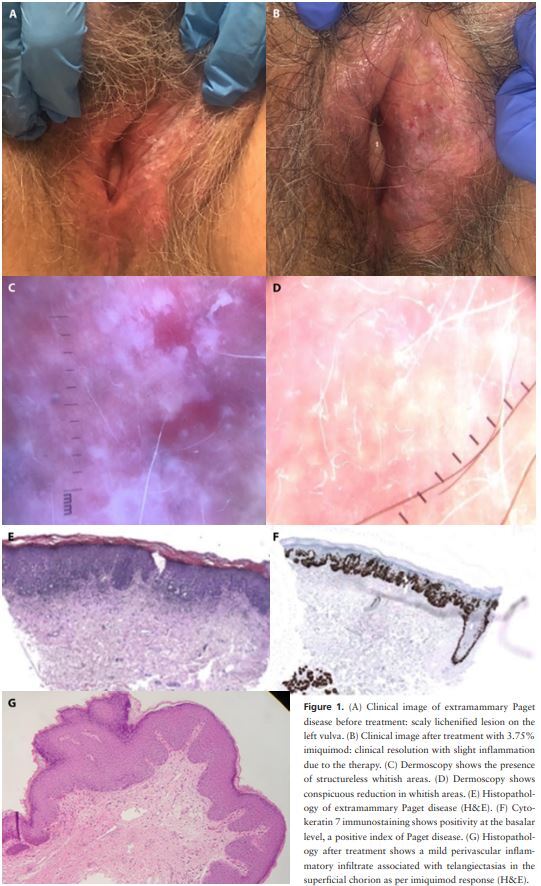
(A) Clinical image of extramammary Paget disease before treatment: scaly lichenified lesion on the left vulva. (B) Clinical image after treatment with 3.75% imiquimod: clinical resolution with slight inflammation due to the therapy. (C) Dermoscopy shows the presence of structureless whitish areas. (D) Dermoscopy shows conspicuous reduction in whitish areas. (E) Histopathology of extramammary Paget disease (H&E). (F) Cytokeratin 7 immunostaining shows positivity at the basalar level, a positive index of Paget disease. (G) Histopathology after treatment shows a mild perivascular inflammatory infiltrate associated with telangiectasias in the superficial chorion as per imiquimod response (H&E).

We performed 2 surgeries on a 60-year-old woman to remove a lesion on the left vulva. A biopsy was performed and was in line with the clinical picture (Figure 1). Histopathological examination and immunohistochemical profile corroborated a diagnosis of EMPD (Figure 1, E and F). We prescribed imiquimod 3.75% following the dosage guidelines: once daily for 15 days, subsequently interrupting therapy for 15 days, and again once a day for other 15 days. After about 5 months she repeated another therapy session and achieved good clearance of the lesions without obvious side effects or post-surgical sequelae. There was clinical and dermoscopic improvement of the lesion after treatment (Figure 1D). After treatment, a biopsy was performed to confirm EMPD absence (Figure 1G).

We used the topical disease approach for this case of CK7+ low progression EMPD to avoid another surgery and the high psychological and physical impact it might have, reclaim the previously excised region, and minimize the use of drugs in order to reduce local adverse events.

## Conclusions

Topical imiquimod 3.75% is an excellent therapeutic tool for CK7+ EMPD in cases when surgery is not recommended. Imiquimod could also be used in cases of recurrence or in a post-adjuvant therapy regime to avoid the risk of disease relapse, or to avoid permanent anogenital mutilation and functional impairment after surgery. More studies are required to evaluate tailor-made therapies based on the EMPD histopathological phenotype, stains and molecular markers.
